# The influence of an accredited pediatric emergency medicine program on the management of pediatric pain and anxiety

**DOI:** 10.1186/s13584-018-0211-6

**Published:** 2018-03-21

**Authors:** Tali Capua, Zohar Bar Kama, Ayelet Rimon

**Affiliations:** Pediatric Emergency Medicine, Dana-Dwek Children’s Hospital, Tel Aviv Sourasky Medical Center, Sackler Faculty of Medicine, Tel Aviv University, 6 Weizman Street, 64239 Tel Aviv, Israel

**Keywords:** Pediatric, Emergency department, Procedural pain, Anxiety, Analgesia

## Abstract

**Background:**

The emergency department (ED) setting is an environment where children may experience intense physical pain and emotional stress.

This study sought to determine the availability of pain and anxiety management practices in all Israeli emergency departments which accept children, specifically looking for differences between accredited pediatric emergency medicine departments and others.

**Methods:**

A cross-sectional survey of all Israeli emergency departments that accept children was performed. One person at each institution was approached to complete the survey. Data were collected between May and June 2016 using an electronic survey tool.

**Results:**

Responses were collected from 21 of 22 hospitals (95% response rate). Commonly available in all types of emergency departments were nurse ordered analgesia, medical clowns (in 95% of the hospitals), topical analgesia and oral sucrose solution. The accredited pediatric emergency medicine departments showed a tendency for more frequent use of all pharmacologic methods for pain and anxiety relief, specifically oxycodone and ketamine.

**Conclusions:**

Overall, Israeli emergency departments have similar access to pharmacologic and non-pharmacologic pain and anxiety management strategies in children, but gaps still exist, especially where not all attending physicians are pediatric emergency medicine trained. We suggest that certified pediatric emergency medicine physicians should advise all emergency departments that accept children to promote the use of the various methods of pain and anxiety reduction.

## Background

The emergency department (ED) setting is an environment where children may experience intense physical pain and emotional stress [1]. There are many factors that may contribute to pain and anxiety in the ED. The child’s symptoms which led to the ED visit are typically a primary cause; however environmental stimuli, physical discomfort and loss of control lead to significant anxiety in children, sometimes precluding successful completion of what is medically indicated [2]. Unfortunately, many barriers encountered when trying to alleviate these issues [3]. Studies show there is under-treatment of pain in the ED of classically painful conditions such as fractures [4–6]. Several studies show that there are effective strategies to reduce pain and anxiety among children in the ED [7–9]_._

The pediatric emergency physician is the primary advocate for treatment of children’s pain and anxiety and for the safe and appropriate use of procedural sedation. Anxiety and pain are intricately interrelated. Trained pediatric ED medical personal are aware that the approach to pain must include an appreciation of anxiety, and vice versa. Encouragingly, improvements in the recognition and treatment of pain in children have led to changes in the approach to pain management for acutely ill and injured pediatric patients [10]. Policy statements of pain and pediatric and emergency societies endorse the appropriate treatment of children’s pain as a key part of ED clinical care [11, 12]. Triage and nursing protocols can identify patients with pain early in their emergency visits, facilitating early treatment of pain and consequently reducing anxiety [13].

In 2007, pediatric emergency medicine (PEM) was officially recognized as a medical specialty in Israel. In 2010, the first certification examinations were held in Israel for physicians who had undergone PEM training. Approximately half a million ED visits are reported in Israel for children younger than 15 across the 22 public hospitals that accept children (http://www.health.gov.il/PublicationsFiles/Malrad.pdf). Israel PEM physicians were historically employed in ten designated pediatric EDs, but in correspondence with the increase in the number of PEM trained physicians, general and pediatric hospitals increasingly staff PEM specialists in their EDs.

To our knowledge, no study has systematically surveyed EDs across Israel to determine which resources and practices are currently in place, to reduce pain and anxiety for children. The objectives of the present study were to assess current resources and strategies in Israeli EDs for pain and anxiety management in children, and to determine which techniques are most widely implemented. In search of gaps that could be easy to address, we further aimed to compare the methods used in hospitals with accredited pediatric EDs to the methods used in hospitals which either have a pediatric ED that is not accredited or a general ED the accepts children.

## Methods

### Study design and population

We conducted a cross-sectional survey to assess pain and anxiety management practices and available resources in all Israeli EDs that accept children. The institutional review board at Tel Aviv Sourasky Medical Center reviewed the study protocol and determined that return of the survey was implied consent for participation.

### Survey content and administration

We developed an online survey to determine the availability of specific procedures and medications for pain or anxiety management, as well as the perceived frequency each specific procedure or medication is used. Our research team compiled a list of all types of pharmacologic and non-pharmacologic pain management strategies by consulting with the research literature and with experts within the field of pediatric pain. We used a five-point Likert scale to assess the frequency of use of the management strategies (from very frequently to very rarely). Once we developed the online version of the survey, two PEM specialists and two head nurses completed it to assess its relevance, its usability, and the total time for completion. Then, we incorporated the feedback from these participants into subsequent modifications.

We contacted the head nurse of each ED or pediatric ED in all public hospitals across the country between May and June, 2016, and asked the head nurse to complete the online survey on Google forms. If a survey was not initially returned, we made at least two attempts at a telephone call follow-up and sent two email reminders. In addition, we contacted the directors of the EDs to encourage participation.

### Data analysis

We collected data on the Google docs platform and imported it for analysis using SPSS version 17 (IBM Corporation, USA). We divided the hospitals into two groups: Accredited PEM service (aPEM) vs. non-accredited PEM (nPEM). The latter are hospitals that either have a non-accredited pediatric ED or a general ED that accepts pediatric patients and is staffed with pediatricians and emergency medicine specialists. We used descriptive statistics were used to present categorical variables as proportions.

## Results

Of the 22 hospitals surveyed, 21 (95%) survey instruments were returned. Responders were 10 aPEM hospitals and 11 nPEM hospitals. All geographic areas of the country were represented.

### Education and guidelines on pain management

Eighty percent of aPEM hospitals and 55% of nPEM hospitals reported having a poster explaining pain and patients’ rights for analgesics, with lower availability of a pamphlet (40% in aPEM hospitals vs. 27% in nPEM hospitals).

### Non-pharmacologic pain and anxiety management

Positive response rates for non-pharmacologic methods are described in Table 1. No statistically significant difference was demonstrated between ED types.

**Table 1:** Comparison of non-pharmacologic pain and anxiety management

	Total *n* = 21	aPEM *n* = 10	nPEM *n* = 11	*p* value*
Child life specialist	10%	10%	9%	0.9392
Medical clown	95%	100%	91%	0.3429
Video games	24%	20%	27%	0.7130
Tablet	19%	20%	18%	0.9093
Wall decorations	95%	100%	91%	0.3429
Music in procedure room	67%	60%	73%	0.5375
Soap bubbles	52%	70%	36%	0.1284
Ability to choose position during procedure	67%	50%	82%	0.1292
Ability to sit on parent during procedure	76%	70%	82%	0.5286
Pappouse board	10%	10%	9%	0.9392
Blood drawing chair	5%	–	9%	0.3429
Vibration tool	–	–	–	NS
Hot or cold packs	10%	10%	9%	0.9392

*NS* No significance

*Chi-squared test comparing aPEM with nPEM

All aPEM hospitals and most nPEM hospitals (91%) reported having access to medical clowns. In contrast, only two hospitals (10%) reported having use of a certified child-life specialist (CCLS) (one aPEM hospital, one nPEM hospital).

Most EDs reported options available for positioning, which mainly meant patient ability to choose position during procedures (50% aPEM, 82% nPEM) and allowing patients to sit on parent’s lap during procedures (70% aPEM, 82% nPEM). Access to a papoose binding tool (one aPEM and one nPEM) and a specialized desk for blood drawing (one nPEM) were infrequently found.

Distraction techniques were not frequently used, except that most EDs reported having decorations on the walls of the procedure room (all aPEM and almost all nPEM (91%). Devices for changes in touch and temperature as a non-pharmacologic distraction are barely available.

### Pharmacologic pain and anxiety management

Pharmacological analgesics and anxiolytics available are described in Table 2. No statistically significant difference was demonstrated between ED types.

**Table 2:** Comparison of pharmacologic pain and anxiety management

	Total *n* = 21	aPEM *n* = 10	nPEM *n* = 11	*p* value*
Ethyl Chloryl analgesic spray	62%	70%	55%	0.4897
EMLA	100%	100%	100%	NS
LET	91%	90%	91%	0.9392
Lidocaine jelly	100%	100%	100%	NS
Oral sucrose solution	90%	90%	91%	0.9392
Nurse ordered analgesia				
Standing order for oral paracetamol	95%	100%	91%	0.3429
Standing order for oral ibuprofen	90%	100%	82%	0.1689

*NS* No significance

*Chi-squared test comparing aPEM with nPEM

Almost all aPEM (90%) and nPEM (91%) offer oral sucrose solution for sucking during neonatal procedures. Eutectic mixture of local anesthetics (EMLA) is the only topical anesthetic available for intact skin in Israel, and it is used by all EDs for blood draws, intravenous access and lumbar puncture and abscess. Lidocaine/epinephrine/tetracaine (LET) gel is widely used for application before laceration repair. All centers reported using lidocaine topical jelly to lubricate the catheter prior to urine catheterization.

**Fig. 1:**
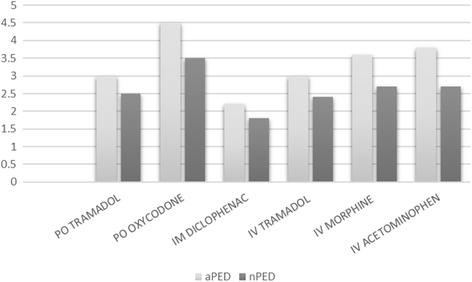
Frequency of use of second line analgesics

Almost all institutions reported using the nationally accredited protocol that gives nurses the ability to order analgesics without a physician’s order (*n* = 19). All aPEM nurses are able to provide paracetamol and ibuprofen, with a reduced ability in nPEMs that is not statistically significant. Variable use of stronger analgesics is demonstrated in Fig. 1, with oxycodone being the most frequently used medication. Intravenous acetaminophen is available in all hospitals.

**Fig. 2:**
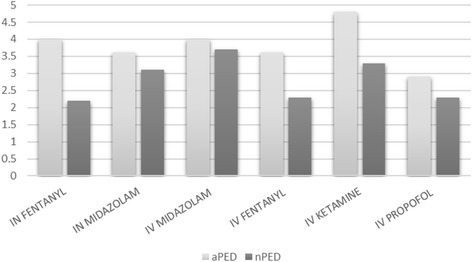
Frequency of use of sedation medications

Pediatric sedation is practiced in both types of EDs. Half of the aPEM have the ability to use nitrous oxide gas for sedation while only one nPEM reported having use. Use of the following analgesics/sedatives was found: intranasal fentanyl or midazolam, intravenous fentanyl, midazolam, ketamine or propofol. While all are widely available, Fig. 2 demonstrates the variability in frequency of use, with ketamine showing a tendency towards being the most frequently use medication in aPEM. No comparison reached a statistically significant level of difference.

## Discussion

This study describes the variability of available strategies and resources used for pain and anxiety relief in Israeli EDs and identifies the differences between aPEM and nPEM. It also highlights opportunities for improvements. While good strategies are reported available in most EDs, there are some important deficits that can be easily implemented to ensure a more positive experience for children in all EDs. For example, the use of hot/cold packs and the use of vibration tools are seldom used in Israel. These quick, low-cost tools could enhance children’s perception to pain significantly when undergoing intravenous access or blood tests. The use of bubbles and audio-visual aids such as tablets also improve a child’s ability to endure painful procedures in the ED.

Medical clowns had a very high presence in the all EDs but unfortunately only two centers have a CCLS. A CCLS aids by providing developmentally appropriate information, giving opportunities for emotional expression by patients and their families and helping establish trust with the pediatric health care team (http://www.childlife.org/child-life-profession/the-case-for-child-life). The use of a CCLS has been shown to improve behavioral stress during procedures [14]. Adding a CCLS in EDs was recommended by the American Academy of Pediatrics as a key resource to help children and their parents during their ED experience, reducing patient anxiety and pain and educating the ED staff [15]. We recommend adding a CCLS to emergency departments to help with workflow and patient satisfaction.

The ministry of health has issued guidelines for nurse-initiated analgesia [16]. This initiative proved to be successful, since almost all institutions reported using the nationally accredited protocol.

Interestingly we found that aPEMs showed a tendency for more frequent use of all pharmacologic methods for pain and anxiety relief when compared to nPEMs. This is true for all modalities. It is possible, that all institutions have the appropriate medications available to use in children, but that the presence of PEM attending physicians better ensure the use of these pharmacologic pain and anxiety relief strategies.

Allowing autonomy for positioning during a procedure may be important tool in decreasing pain and anxiety. A recent study found that sitting a young child upright for venipuncture provided higher satisfaction and did not statistically alter the number of IV attempts [16]. aPEMs tended to offer less choice of positioning during procedures. This may be because they tended to offer other forms of relief or relied on more pharmacologic forms of pain and anxiety reduction. Allowing different positions for procedures including having the parent hold the child should be offered in the emergency room setting.

It should be noted that while EMLA is the only topical anesthetic available for intact skin in Israel, LMX (topical liposomal lidocaine) is another formulation available in other countries. EMLA vasoconstricts and reaches peak activity at one hour while LMX does not vasoconstrict and reaches its peak much earlier at 30 min [17]. In the future, LMX should be considered as it may aid in a more efficient experience for patients and staff.

Furthermore, while ethyl chloride analgesic spray is available for intravenous access, a recent review found that it does not help reduce pain during blood draws and its application causes pain. It is therefore not recommended for use in children [18]. Efforts should be made to make staff members aware of these developments in pharmacologic pain relief.

The present study had several limitations. First, despite surveying all Israeli EDs which accept children, the compared groups were small. As a result, the low statistical power has a reduced chance of detecting a true difference in practice.

Second, we relied only on the report of a single individual at each center. While this individual was identified as being the most knowledgeable, answers may not be perfectly representative of the center’s practice. When asked about frequency of use of a strategy, this individual may have reflected his own preferences. This survey was planned as a qualitative account of pediatric pain and anxiety management; however, we would like to study quantitative data on medication use in EDs. We are currently in the process of collecting data from the hospital pharmacies, to better quantify medication use in EDs.

## Conclusions

The present study described strategies and resources available to physicians in the management of pediatric pain and anxiety at Israeli EDs. It also helped to identify possible areas for improvement at individual centers, with an emphasis on the difference between aPEM hospitals and nPEM hospitals. Every ED that accepts children should be prepared to manage pain, anxiety and distress in sick and injured children. Certified PEM physicians should advise all institutions, to promote the use of the various methods described in this survey. We have demonstrated that all EDs have access to pharmacologic and non-pharmacologic pain and anxiety management strategies, but that their frequency of use of some pharmacological analgesics may be influenced by the presence of aPEM.
